# Crystal structure of 1,3-diallyl-1,3,3a,4,7,7a-hexa­hydro-4,7-methano-2-benzo­thio­phene 2,2-dioxide

**DOI:** 10.1107/S1600536814022053

**Published:** 2014-10-15

**Authors:** Sambasivarao Kotha, Rama Gunta

**Affiliations:** a Department of Chemistry, Indian Institute of Technology–Bombay, Powai, Mumbai 400 076, India

**Keywords:** crystal structure, allyl­ation, norbonene derivatives, sulfones

## Abstract

The title compound C_15_H_20_O_2_S, was identified as a product of di­allyl­ation of the *meso*-isomer of the corresponding norbornene sulfone, and it is an achiral compound. The five-membered heterocycle adopts an envelope conformation with the S atom deviating by 0.795 (3) Å from the other atoms of the ring (r.m.s. deviation = 0.0131). Both allyl groups are *anti*-oriented relative to the S atom but their double bonds are directed in opposite directions relative to the plane of the heterocycle.

## Related literature   

For related functionalized sulfones, see: Bloch & Abecassis (1982 ▶, 1983 ▶); Bloch *et al.* (1983 ▶, 1984 ▶); Yamada *et al.* (1983 ▶). For the synthesis of the precursor, see: Bloch & Abecassis (1982 ▶). For sulfones as latent diene equivalents, see: Fringuelli & Taticchi (1990 ▶). For X-ray crystal data of related bi­cyclo­[2.2.1]compounds, see: Birney *et al.* (2002 ▶). For literature on sulfones, see: Bhat (1994 ▶); Fielder *et al.* (2000 ▶); Nakayama *et al.* (1997 ▶). For bond lengths in related structures, see: Chandrasekhar (1992 ▶); Pool & White (2000 ▶).
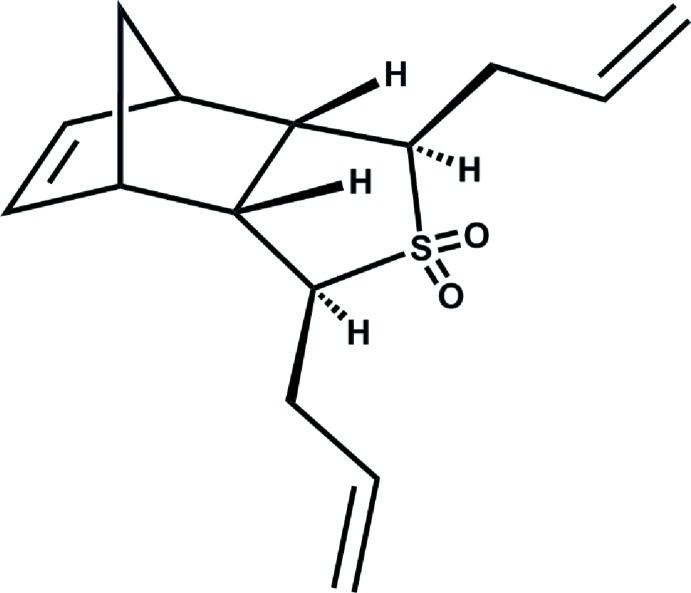


## Experimental   

### Crystal data  

C_15_H_20_O_2_S
*M* *_r_* = 264.37
Monoclinic, 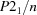

*a* = 12.4412 (17) Å
*b* = 8.8472 (13) Å
*c* = 12.738 (2) Å
β = 97.069 (8)°
*V* = 1391.4 (4) Å^3^
*Z* = 4
Mo *K*α radiation
μ = 0.23 mm^−1^
*T* = 100 K
0.27 × 0.22 × 0.11 mm

### Data collection  

Rigaku Saturn724 diffractometer
Absorption correction: numerical (*NUMABS*; Rigaku, 1999 ▶) *T*_min_ = 0.957, *T*_max_ = 0.976
20804 measured reflections
3702 independent reflections
3231 reflections with *I* > 2σ(*I*)
*R*_int_ = 0.097

### Refinement  

*R*[*F* ^2^ > 2σ(*F* ^2^)] = 0.069
*wR*(*F* ^2^) = 0.141
*S* = 1.13
3702 reflections
163 parameters
H-atom parameters constrained
Δρ_max_ = 0.46 e Å^−3^
Δρ_min_ = −0.46 e Å^−3^

### 

Data collection: *CrystalClear-SM Expert* (Rigaku, 2013 ▶); cell refinement: *CrystalClear-SM Expert*; data reduction: *CrystalClear-SM Expert*; program(s) used to solve structure: *SHELXS97* (Sheldrick, 2008 ▶); program(s) used to refine structure: *SHELXL97* (Sheldrick, 2008 ▶); molecular graphics: *CrystalStructure* (Rigaku, 2010 ▶); software used to prepare material for publication: *CrystalStructure*.

## Supplementary Material

Crystal structure: contains datablock(s) I. DOI: 10.1107/S1600536814022053/ld2131sup1.cif

Structure factors: contains datablock(s) I. DOI: 10.1107/S1600536814022053/ld2131Isup2.hkl

**Fig. 1.**
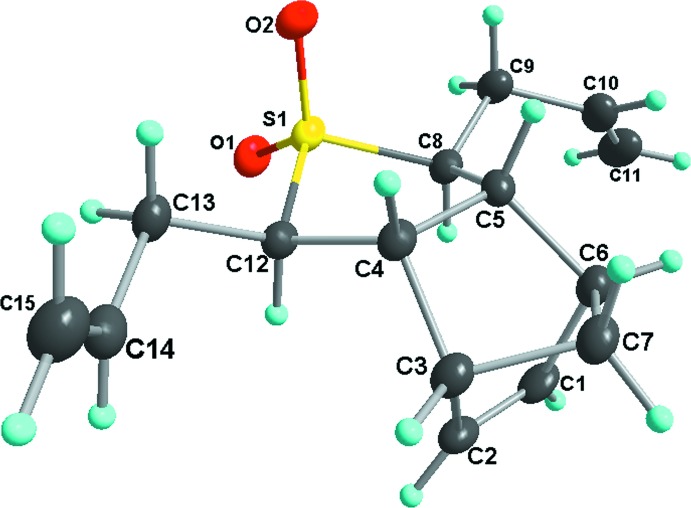
The stucture of the title compound showing labeling of non-H atoms.

. DOI: 10.1107/S1600536814022053/ld2131fig1.tif
The stucture of the title compound showing labeling of non-H atoms.

CCDC reference: 1027850

Additional supporting information:  crystallographic information; 3D view; checkCIF report

## supplementary crystallographic information

### S1. Comments

Alkyl­ated sulfone derivatives have found useful application in the synthesis of (*E*)-9,11-dodecadien-l-y1 acetate, a sex pheromone of the red-bollworm moth (Bloch & Abecassis, 1982). Moreover, sulfones (Bhat, 1994; Fielder *et al.*, 2000; Nakayama *et al.*, 1997) are latent diene (Fringuelli & Taticchi, 1990) equivalents. In view of the applications of various alkyl and other functionalized sulfone derivatives (Bloch & Abecassis, 1983; Bloch *et al.*, 1983; Yamada *et al.*, 1983), we have synthesized the title compound, C_15_H_20_O_2_S (Figure 1), which is a non-chiral meso compound (i.e. inter­nal recemate). The corresponding mono-allyl­ated sulfone has been reported previously (Bloch *et al.*, 1984). The starting sulfone was allyl­ated with allyl bromide using *n*-BuLi as a base at –75 °C to room temperature for 25 h to furnish the desired di-allyl­ated sulfone 1 in 80% yield along with the mono-allyl­ated sulfone in 10% yield. After recrystallization from a mixture of hexane-di­chloro­methane (3:1), we obtained monoclinic crystals of the compound 1. The single-crystal X-ray study of the compound 1 clearly indicates that the di-allyl­ation has been occurred at α,α^'^-positions of the sulfone functionality with the allyl groups *cis*-positioned relatively to each other (see Figure 1). This stereoselectivity can be explained by stronger sterical hindrances at the *endo* face of the sulfone ring for the approaching electrophile (allyl bromide) as compared with those at its *exo* face. The title compound exhibits single bonds Csp^3^—Csp^3^ elongated up to 1.576 (3) Å [C4—C5]. The bond lengths C3—C4 of 1.559 (3) Å and C5—C6 of 1.568 (3) Å are also longer than that of the standard average Csp^3^— Csp^3^ single bond [1.54 Å] (Chandrasekhar, 1992). The bond angle C3—C7—C6 of 94.14 (17) Å is contracted the most relative to the standard tetra­hedral value of 109.5°. The angle C13—C14—C15 is found to be the most expanded one at 125.1 (2)°. The five-membered heterocycle has an envelope conformation, in which four C atoms are in the same plane (C8—C5—C4—C12) and SO_2_ group deviates from it. Two allylic double bonds are oriented in opposite direction to each other. Previously, Pool and White (2000) have also reported that the average lengths of C—C bonds corresponding to C3—C4 and C5—C6 in our structure in the similar bicyclic cyclo­hexene derivatives are also significantly longer (by 0.02 Å) than the corresponding C—C bond distances for the saturated bicyclic cyclo­hexane derivatives. Later, Birney and co-workers (Birney *et al.*, 2002) have studied the X-ray crystal data of bi­cyclo­[2.2.1]moiety containing compounds in order to estimate their *retro*-Diels–Alder reactivity.

### S2. Experimental Section

Melting points were recorded on Veego melting point apparatus and are uncorrected. Nuclear Magnetic Resonance (NMR) spectra were recorded on a Bruker (Avance III^TM^ 500) spectrometer operated at 500 MHz for ^1^H and 125.7 MHz for ^13^C nuclei. The high-resolution mass spectrometric (HRMS) measurements were carried out using Bruker (Maxis Impact) instrument. Infrared (IR) spectrum of solid sample was recorded as KBr pellets on Nicolet Impact-400 FT IR spectrometer.

### S2.1. Synthesis and Crystallization of Compound_1

The solution of the precursor sulfone (600 mg, 3.26 mmol) in anhydrous THF (15 mL) was cooled to –75 °C under nitro­gen (N_2_). To this solution, *n*-BuLi (4.90 mL, 2.4 equiv, 1.6 M solution in hexanes) was added in dropwise manner and the reaction mixture was stirred for 30 min. Later, allyl bromide (0.83 mL, 9.77 mmol) was added slowly and the stirring continued at the same temperature for 2 h. Then, the reaction mixture was allowed to raise to the room temperature and the stirring continued for 22 h more. At the conclusion of the reaction (TLC monitoring), the reaction mixture was quenched with water (5 mL) and the solvent was removed under reduced pressure. Then, the resulting residue was extracted with di­ethyl ether (3 × 30 mL). The combined organic layers were washed with brine (2 × 20 mL) and dried over anhydrous Na_2_SO_4_. The solvent was removed under reduced pressure and the crude product was purified by silica gel column chromatography using ethyl acetate-petroleum ether (1:9) as an eluent to afford the di-allyl­ated sulfone 1 (690 mg, 80%) as a white crystalline solid. A further elution with ethyl acetate-petroleum ether (2:8) delivered the previously known mono-allyl­ated sulfone (75.40 mg, 10%) as a pale yellow liquid. ^1^H and ^13^C NMR spectroscopic data of the mono-allyl­ated sulfone was compared with the literature report (Bloch *et al.*, 1984) and found to be identical. After purification the title compound was recrystallized from a mixture of hexane-di­chloro­methane (3:1) by slow evaporation to get the colourless crystals. *R_f_* = 0.91 (20% ethyl acetate in petroleum ether); mp: 348.15–349.15 K. ^1^H NMR (500 MHz, CDCl_3_): δ (ppm) = 6.20 (br s, 2H), 5.88–5.80 (m, 2H), 5.23 (dd, *J* = 16.9, 1.2 Hz, 2H), 5.16 (d, *J* = 10.0 Hz, 2H), 2.99 (br s, 2H), 2.77–2.72 (m, 2H), 2.45–2.40 (m, 2H), 2.37–2.30 (m, 4H), 1.67 (d, *J* = 8.8 Hz, 1H), 1.39 (d, *J* = 8.8 Hz, 1H); ^13^C NMR (125.7 MHz, CDCl_3_): δ (ppm) = 136.9, 133.6, 118.6, 62.5, 49.8, 45.6, 45.3, 30.8; HRMS (ESI, Q-ToF) *m/z*: calculated for C_15_H_20_NaO_2_S [M+Na]^+^ 287.1076, found: 287.1077; IR (neat): *υ*_max_ = 3021, 2978, 1640, 1443, 1306, 1216, 1123 cm^-1^.

### S2.2. Refinement

Crystal data, data collection and structure refinement details are summarized in Table 1. All H atoms were placed in their geometrically calculated positions and refined using a riding model with C—H distances of 0.95 Å for all H atoms bound to *sp*^2^ C atoms and 1.00 Å for all others. U_iso_(H) = *x*U_eq_(C), where *x* = 1.5 for allylic methyl­idene [═C(11,15)H_2_] H atoms and 1.2 for all other H atoms. The positions of allylic methyl­idene and non-allylic methine [═C(1,2)H—] H atoms were calculated using the SHELXL-97 instructions AFIX 93 and AFIX 43 respectively (Sheldrick, 2008).

### Crystal data

**Table:**

C_15_H_20_O_2_S	*F*(000) = 568
*M**_r_* = 264.37	*D*_x_ = 1.262 Mg m^−^^3^
Monoclinic, *P*2_1_/*n*	Melting point = 349.15–348.15 K
Hall symbol: -P 2yn	Mo *K*α radiation, λ = 0.71075 Å
*a* = 12.4412 (17) Å	Cell parameters from 3728 reflections
*b* = 8.8472 (13) Å	θ = 3.2–29.1°
*c* = 12.738 (2) Å	µ = 0.23 mm^−^^1^
β = 97.069 (8)°	*T* = 100 K
*V* = 1391.4 (4) Å^3^	Prism, colourless
*Z* = 4	0.27 × 0.22 × 0.11 mm

### Data collection

**Table:**

Rigaku Saturn724 diffractometer	3702 independent reflections
Radiation source: fine-focus sealed tube	3231 reflections with *I* > 2σ(*I*)
Graphite monochromator	*R*_int_ = 0.097
Detector resolution: 7.111 pixels mm^-1^	θ_max_ = 29.2°, θ_min_ = 3.2°
ω scans	*h* = −16→16
Absorption correction: numerical (*NUMABS*; Rigaku, 1999)	*k* = −12→12
*T*_min_ = 0.957, *T*_max_ = 0.976	*l* = −17→17
20804 measured reflections	

### Refinement

**Table:**

Refinement on *F*^2^	Primary atom site location: structure-invariant direct methods
Least-squares matrix: full	Secondary atom site location: difference Fourier map
*R*[*F*^2^ > 2σ(*F*^2^)] = 0.069	Hydrogen site location: inferred from neighbouring sites
*wR*(*F*^2^) = 0.141	H-atom parameters constrained
*S* = 1.13	*w* = 1/[σ^2^(*F*_o_^2^) + (0.0388*P*)^2^ + 1.1426*P*] where *P* = (*F*_o_^2^ + 2*F*_c_^2^)/3
3702 reflections	(Δ/σ)_max_ < 0.001
163 parameters	Δρ_max_ = 0.46 e Å^−^^3^
0 restraints	Δρ_min_ = −0.46 e Å^−^^3^

### Special details

**Table:**

Geometry. All e.s.d.'s (except the e.s.d. in the dihedral angle between two l.s. planes) are estimated using the full covariance matrix. The cell e.s.d.'s are taken into account individually in the estimation of e.s.d.'s in distances, angles and torsion angles; correlations between e.s.d.'s in cell parameters are only used when they are defined by crystal symmetry. An approximate (isotropic) treatment of cell e.s.d.'s is used for estimating e.s.d.'s involving l.s. planes.
Refinement. Refinement of *F*^2^ against ALL reflections. The weighted *R*-factor *wR* and goodness of fit *S* are based on *F*^2^, conventional *R*-factors *R* are based on *F*, with *F* set to zero for negative *F*^2^. The threshold expression of *F*^2^ > σ(*F*^2^) is used only for calculating *R*-factors(gt) *etc*. and is not relevant to the choice of reflections for refinement. *R*-factors based on *F*^2^ are statistically about twice as large as those based on *F*, and *R*- factors based on ALL data will be even larger. All non-hydrogen atoms are refined anisotropically and all hydrogen atoms are refined using riding model.

### Fractional atomic coordinates and isotropic or equivalent isotropic displacement parameters (Å^2^)

**Table:**

	*x*	*y*	*z*	*U*_iso_*/*U*_eq_	
S1	0.13583 (4)	0.04117 (7)	−0.17128 (4)	0.01977 (15)	
O1	0.17802 (13)	0.0793 (2)	−0.26827 (12)	0.0266 (4)	
O2	0.02888 (12)	0.09616 (19)	−0.15801 (13)	0.0248 (4)	
C1	0.37378 (18)	−0.1394 (3)	0.04960 (18)	0.0255 (5)	
H1A	0.4435	−0.0971	0.0464	0.031*	
C2	0.33509 (17)	−0.2638 (3)	0.00116 (18)	0.0233 (5)	
H2A	0.3715	−0.3258	−0.0441	0.028*	
C3	0.22217 (18)	−0.2896 (3)	0.03084 (18)	0.0227 (5)	
H3A	0.1948	−0.3958	0.0236	0.027*	
C4	0.14638 (17)	−0.1664 (3)	−0.02595 (17)	0.0199 (4)	
H4A	0.0713	−0.1818	−0.0076	0.024*	
C5	0.19392 (17)	−0.0161 (3)	0.02751 (17)	0.0203 (5)	
H5A	0.1374	0.0338	0.0650	0.024*	
C6	0.28701 (18)	−0.0765 (3)	0.11112 (18)	0.0231 (5)	
H6A	0.3126	−0.0058	0.1702	0.028*	
C7	0.23523 (18)	−0.2246 (3)	0.14310 (17)	0.0240 (5)	
H7A	0.2844	−0.2853	0.1936	0.029*	
H7B	0.1651	−0.2086	0.1708	0.029*	
C8	0.22743 (17)	0.0912 (3)	−0.05690 (17)	0.0214 (5)	
H8A	0.3024	0.0636	−0.0706	0.026*	
C9	0.22503 (19)	0.2605 (3)	−0.03435 (19)	0.0267 (5)	
H9A	0.2466	0.3170	−0.0955	0.032*	
H9B	0.1504	0.2912	−0.0245	0.032*	
C10	0.3005 (2)	0.2993 (3)	0.0631 (2)	0.0303 (6)	
H10A	0.2786	0.2739	0.1298	0.036*	
C11	0.3942 (2)	0.3655 (3)	0.0626 (3)	0.0397 (7)	
H11A	0.4187	0.3924	−0.0027	0.060*	
H11B	0.4379	0.3867	0.1274	0.060*	
C12	0.14238 (17)	−0.1576 (3)	−0.14620 (16)	0.0191 (4)	
H12A	0.2121	−0.1974	−0.1667	0.023*	
C13	0.04834 (18)	−0.2400 (3)	−0.21087 (18)	0.0226 (5)	
H13A	−0.0210	−0.2029	−0.1898	0.027*	
H13B	0.0489	−0.2167	−0.2868	0.027*	
C14	0.05525 (18)	−0.4070 (3)	−0.19523 (18)	0.0257 (5)	
H14A	0.1178	−0.4568	−0.2139	0.031*	
C15	−0.0184 (2)	−0.4906 (3)	−0.1576 (2)	0.0321 (6)	
H15A	−0.0822	−0.4450	−0.1380	0.048*	
H15B	−0.0080	−0.5966	−0.1500	0.048*	

### Atomic displacement parameters (Å^2^)

**Table:**

	*U*^11^	*U*^22^	*U*^33^	*U*^12^	*U*^13^	*U*^23^
S1	0.0159 (3)	0.0258 (3)	0.0175 (3)	−0.0011 (2)	0.00159 (19)	0.0028 (2)
O1	0.0258 (8)	0.0346 (10)	0.0197 (8)	−0.0032 (7)	0.0045 (6)	0.0060 (7)
O2	0.0173 (8)	0.0308 (9)	0.0258 (9)	0.0009 (6)	0.0013 (6)	0.0032 (7)
C1	0.0146 (10)	0.0360 (14)	0.0253 (12)	0.0017 (9)	−0.0001 (8)	0.0075 (10)
C2	0.0194 (10)	0.0302 (13)	0.0205 (11)	0.0071 (9)	0.0027 (8)	0.0036 (10)
C3	0.0201 (11)	0.0255 (12)	0.0220 (11)	0.0001 (9)	0.0001 (8)	0.0031 (9)
C4	0.0145 (9)	0.0264 (12)	0.0188 (10)	−0.0009 (8)	0.0023 (8)	0.0010 (9)
C5	0.0161 (10)	0.0274 (12)	0.0174 (10)	0.0008 (8)	0.0023 (8)	−0.0026 (9)
C6	0.0204 (11)	0.0290 (13)	0.0192 (11)	0.0006 (9)	−0.0010 (8)	0.0003 (9)
C7	0.0203 (11)	0.0328 (13)	0.0188 (11)	0.0003 (9)	0.0019 (8)	0.0030 (10)
C8	0.0158 (10)	0.0274 (12)	0.0206 (11)	−0.0003 (8)	0.0007 (8)	−0.0008 (9)
C9	0.0234 (11)	0.0269 (13)	0.0285 (12)	0.0026 (9)	−0.0018 (9)	−0.0012 (10)
C10	0.0342 (13)	0.0273 (13)	0.0283 (13)	0.0012 (10)	−0.0002 (10)	−0.0022 (11)
C11	0.0337 (14)	0.0323 (15)	0.0504 (17)	−0.0011 (11)	−0.0054 (12)	−0.0059 (13)
C12	0.0162 (10)	0.0240 (11)	0.0173 (10)	−0.0001 (8)	0.0025 (8)	0.0006 (9)
C13	0.0203 (10)	0.0268 (12)	0.0196 (11)	−0.0033 (9)	−0.0016 (8)	0.0005 (9)
C14	0.0214 (11)	0.0314 (13)	0.0238 (12)	−0.0032 (9)	0.0006 (9)	−0.0044 (10)
C15	0.0264 (12)	0.0335 (14)	0.0349 (14)	−0.0044 (10)	−0.0023 (10)	0.0031 (11)

### Geometric parameters (Å, º)

**Table:**

S1—O1	1.4406 (16)	C7—H7A	0.9900
S1—O2	1.4461 (16)	C7—H7B	0.9900
S1—C12	1.788 (2)	C8—C9	1.527 (3)
S1—C8	1.791 (2)	C8—H8A	1.0000
C1—C2	1.323 (3)	C9—C10	1.502 (3)
C1—C6	1.516 (3)	C9—H9A	0.9900
C1—H1A	0.9500	C9—H9B	0.9900
C2—C3	1.516 (3)	C10—C11	1.305 (4)
C2—H2A	0.9500	C10—H10A	0.9500
C3—C7	1.531 (3)	C11—H11A	0.9500
C3—C4	1.559 (3)	C11—H11B	0.9500
C3—H3A	1.0000	C12—C13	1.530 (3)
C4—C12	1.528 (3)	C12—H12A	1.0000
C4—C5	1.576 (3)	C13—C14	1.492 (3)
C4—H4A	1.0000	C13—H13A	0.9900
C5—C8	1.530 (3)	C13—H13B	0.9900
C5—C6	1.568 (3)	C14—C15	1.313 (3)
C5—H5A	1.0000	C14—H14A	0.9500
C6—C7	1.537 (3)	C15—H15A	0.9500
C6—H6A	1.0000	C15—H15B	0.9500
			
O1—S1—O2	117.32 (10)	C3—C7—H7B	112.9
O1—S1—C12	111.75 (10)	C6—C7—H7B	112.9
O2—S1—C12	109.42 (10)	H7A—C7—H7B	110.3
O1—S1—C8	112.16 (10)	C9—C8—C5	117.59 (19)
O2—S1—C8	108.92 (10)	C9—C8—S1	111.73 (16)
C12—S1—C8	95.00 (10)	C5—C8—S1	102.55 (15)
C2—C1—C6	107.8 (2)	C9—C8—H8A	108.2
C2—C1—H1A	126.1	C5—C8—H8A	108.2
C6—C1—H1A	126.1	S1—C8—H8A	108.2
C1—C2—C3	107.7 (2)	C10—C9—C8	110.8 (2)
C1—C2—H2A	126.2	C10—C9—H9A	109.5
C3—C2—H2A	126.2	C8—C9—H9A	109.5
C2—C3—C7	100.44 (18)	C10—C9—H9B	109.5
C2—C3—C4	107.77 (18)	C8—C9—H9B	109.5
C7—C3—C4	99.18 (18)	H9A—C9—H9B	108.1
C2—C3—H3A	115.7	C11—C10—C9	124.5 (3)
C7—C3—H3A	115.7	C11—C10—H10A	117.8
C4—C3—H3A	115.7	C9—C10—H10A	117.8
C12—C4—C3	116.35 (18)	C10—C11—H11A	120.0
C12—C4—C5	110.74 (18)	C10—C11—H11B	120.0
C3—C4—C5	102.46 (17)	H11A—C11—H11B	120.0
C12—C4—H4A	109.0	C4—C12—C13	116.34 (18)
C3—C4—H4A	109.0	C4—C12—S1	102.97 (15)
C5—C4—H4A	109.0	C13—C12—S1	110.95 (15)
C8—C5—C6	116.52 (18)	C4—C12—H12A	108.8
C8—C5—C4	109.88 (17)	C13—C12—H12A	108.8
C6—C5—C4	102.26 (18)	S1—C12—H12A	108.8
C8—C5—H5A	109.3	C14—C13—C12	111.85 (19)
C6—C5—H5A	109.3	C14—C13—H13A	109.2
C4—C5—H5A	109.3	C12—C13—H13A	109.2
C1—C6—C7	99.92 (19)	C14—C13—H13B	109.2
C1—C6—C5	106.71 (18)	C12—C13—H13B	109.2
C7—C6—C5	99.91 (17)	H13A—C13—H13B	107.9
C1—C6—H6A	116.0	C15—C14—C13	125.1 (2)
C7—C6—H6A	116.0	C15—C14—H14A	117.5
C5—C6—H6A	116.0	C13—C14—H14A	117.5
C3—C7—C6	94.14 (17)	C14—C15—H15A	120.0
C3—C7—H7A	112.9	C14—C15—H15B	120.0
C6—C7—H7A	112.9	H15A—C15—H15B	120.0
			
C6—C1—C2—C3	−1.2 (3)	C4—C5—C8—S1	29.21 (19)
C1—C2—C3—C7	−32.1 (2)	O1—S1—C8—C9	77.19 (18)
C1—C2—C3—C4	71.2 (2)	O2—S1—C8—C9	−54.37 (19)
C2—C3—C4—C12	56.1 (2)	C12—S1—C8—C9	−166.88 (16)
C7—C3—C4—C12	160.25 (18)	O1—S1—C8—C5	−155.97 (14)
C2—C3—C4—C5	−64.9 (2)	O2—S1—C8—C5	72.46 (16)
C7—C3—C4—C5	39.31 (19)	C12—S1—C8—C5	−40.04 (15)
C12—C4—C5—C8	−2.9 (2)	C5—C8—C9—C10	60.6 (3)
C3—C4—C5—C8	121.84 (18)	S1—C8—C9—C10	178.75 (17)
C12—C4—C5—C6	−127.26 (18)	C8—C9—C10—C11	105.1 (3)
C3—C4—C5—C6	−2.5 (2)	C3—C4—C12—C13	97.1 (2)
C2—C1—C6—C7	33.9 (2)	C5—C4—C12—C13	−146.51 (19)
C2—C1—C6—C5	−69.7 (2)	C3—C4—C12—S1	−141.38 (16)
C8—C5—C6—C1	−51.2 (3)	C5—C4—C12—S1	−24.95 (19)
C4—C5—C6—C1	68.6 (2)	O1—S1—C12—C4	154.63 (13)
C8—C5—C6—C7	−154.80 (19)	O2—S1—C12—C4	−73.72 (15)
C4—C5—C6—C7	−35.0 (2)	C8—S1—C12—C4	38.37 (15)
C2—C3—C7—C6	49.4 (2)	O1—S1—C12—C13	−80.22 (17)
C4—C3—C7—C6	−60.77 (18)	O2—S1—C12—C13	51.43 (18)
C1—C6—C7—C3	−49.91 (19)	C8—S1—C12—C13	163.51 (16)
C5—C6—C7—C3	59.17 (19)	C4—C12—C13—C14	−65.6 (3)
C6—C5—C8—C9	−92.2 (2)	S1—C12—C13—C14	177.17 (16)
C4—C5—C8—C9	152.19 (19)	C12—C13—C14—C15	120.0 (3)
C6—C5—C8—S1	144.85 (17)		

## References

[bb1] Bhat, S. V. (1994). *J. Indian Inst. Sci.* **74**, 257–276.

[bb2] Birney, D., Lim, T. K., Koh, J. H. P., Pool, B. R. & White, J. M. (2002). *J. Am. Chem. Soc.* **124**, 5091–5099. 10.1021/ja025634f 11982374

[bb4] Bloch, R. & Abecassis, J. (1983). *Tetrahedron Lett.* **24**, 1247–1250.

[bb5] Bloch, R., Abecassis, J. & Hassan, D. (1984). *Can. J. Chem.* **62**, 2019–2024.

[bb6] Bloch, R., Hassan, D. & Mandard, X. (1983). *Tetrahedron Lett.* **24**, 4691–4694.

[bb3] Bloch, R. & Abecassis, J. (1982). *Tetrahedron Lett.* **23**, 3277–3280.

[bb7] Chandrasekhar, J. (1992). *Curr. Sci.* **63**, 114–116.

[bb8] Fielder, S., Rowan, D. D. & Sherburn, M. S. (2000). *Angew. Chem. Int. Ed.* **39**, 4331–4333. 10.1002/1521-3773(20001201)39:23<4331::AID-ANIE4331>3.0.CO;2-3 29711924

[bb9] Fringuelli, F. & Taticchi, A. (1990). In *Dienes in the Diels–Alder Reaction*. New York: John Wiley & Sons.

[bb10] Nakayama, J., Nagasawa, H., Sugihara, Y. & Ishii, A. (1997). *J. Am. Chem. Soc.* **119**, 9077–9078.

[bb11] Pool, B. R. & White, J. M. (2000). *Org. Lett.* **2**, 3505–3507. 10.1021/ol006553w 11082020

[bb12] Rigaku (1999). *NUMABS*. Rigaku Corporation, Tokyo, Japan.

[bb13] Rigaku (2010). *CrystalStructure*. Rigaku Corporation, Tokyo, Japan.

[bb14] Rigaku (2013). *CrystalClear-SM Expert*. Rigaku Corporation, Tokyo, Japan.

[bb15] Sheldrick, G. M. (2008). *Acta Cryst.* A**64**, 112–122. 10.1107/S0108767307043930 18156677

[bb16] Yamada, S., Ohsawa, H., Suzuki, T. & Takayama, H. (1983). *Chem. Lett.* **12**, 1003–1006.

