# Supplementary figures and images for: Crystal structure of 1,3-diallyl-1,3,3a,4,7,7a-hexa­hydro-4,7-methano-2-benzo­thio­phene 2,2-dioxide
Source: Acta Crystallogr Sect E Struct Rep Online. 2014 Oct 15;70(Pt 11):o1163–4. doi: 10.1107/S1600536814022053 (PMC4257250; doi:10.1107/S1600536814022053)

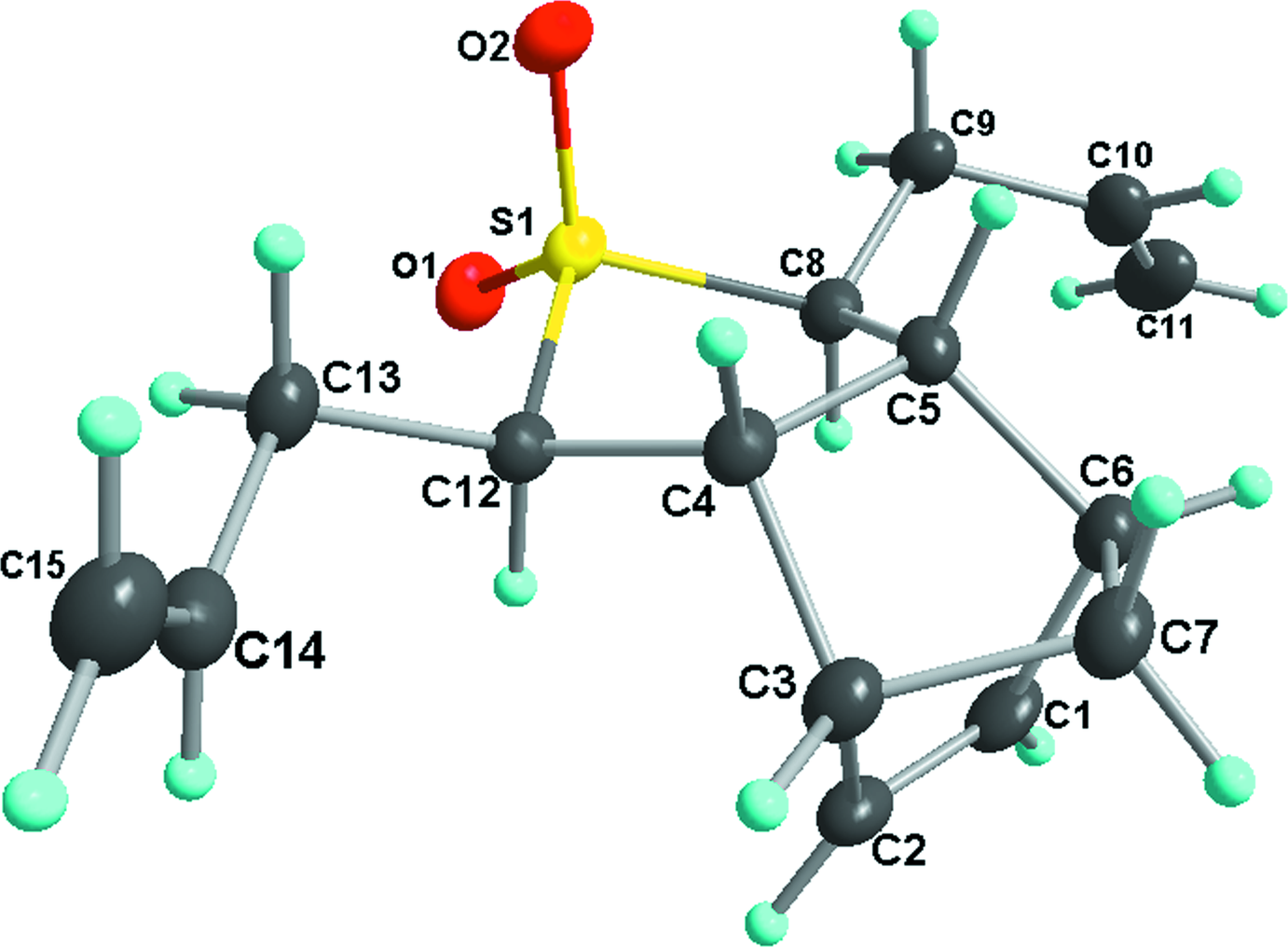

Supplement: Supplementary file 3 [file e-70-o1163-fig1.tif]
